# Synthesis of Magnesium Nickel Boride Aggregates via Borohydride Autogenous Pressure

**DOI:** 10.3390/ma11040480

**Published:** 2018-03-23

**Authors:** Mahboobeh Shahbazi, Henrietta E. Cathey, Ian D. R. Mackinnon

**Affiliations:** Institute for Future Environments and Science and Engineering Faculty, Queensland University of Technology (QUT), Brisbane, QLD 4001, Australia; henrietta.cathey@qut.edu.au (H.E.C.); ian.mackinnon@qut.edu.au (I.D.R.M.)

**Keywords:** ternary metal borides, autogenous pressure synthesis, magnesium nickel boride, microstructures

## Abstract

We demonstrate synthesis of the ternary intermetallic MgNi_3_B_2_ using autogenous pressure from the reaction of NaBH_4_ with Mg and Ni metal powder. The decomposition of NaBH_4_ to H_2_ and B_2_H_6_ commences at low temperatures in the presence of Mg and/or Ni and promotes formation of Ni-borides and MgNi_3_B_2_ with the increase in temperature. MgNi_3_B_2_ aggregates with Ni-boride cores are formed when the reaction temperature is >670 °C and autogenous pressure is >1.7 MPa. Morphologies and microstructures suggest that solid–gas and liquid–gas reactions are dominant mechanisms and that Ni-borides form at a lower temperature than MgNi_3_B_2_. Magnetic measurements of the core-shell MgNi_3_B_2_ aggregates are consistent with ferromagnetic behaviour in contrast to stoichiometric MgNi_3_B_2_ which is diamagnetic at room temperature.

## 1. Introduction

Metal hydrides and metal borides, in particular, Mg-based compounds such as MgH_2_, MgB_2_, and ternary metal varieties that include Ni, are attractive materials for a range of applications including hydrogen storage [1,2,3] as well as electron transport [4,5] and/or electron storage [6]. Metal borides are commonly produced using solid-state reactions with mixed elemental components in a reducing environment at relatively high temperatures [7,8]. Liquid phase methods have been explored for production of nano-scale borides [9,10] but with increased complexity of processing due to the absence of a reactive elemental boron species that can decompose under conditions typical for nano-syntheses. In general, the intrinsic and strong covalent B–B and M–B bonding in boron-based solids [10] requires high temperature conditions to effect syntheses of metal borides.

Typical methods to produce Mg-Ni-B compounds include reacting borides or boron with metals under reducing conditions. For example, mechanical milling of MgB_2_ and Ni precursors followed by sintering at 975 °C with an inert atmosphere [11]; ball milling of Mg, Ni, and B powders with sintering at 800 °C [12]; or heating the elements in a sealed Ta container at 950 °C for seven days [5] results in formation of Mg-Ni-B compounds. In addition, a Mg-Ni-B compound occurs as a reaction layer (ranging from 10 µm to 20 µm thick) on MgB_2_ wires when fabricated with a Ni sheath [13]. The reaction layer forms by migration of Ni into the MgB_2_ core with heat treatments between 800 °C and 960 °C [12].

**Figure 1:**
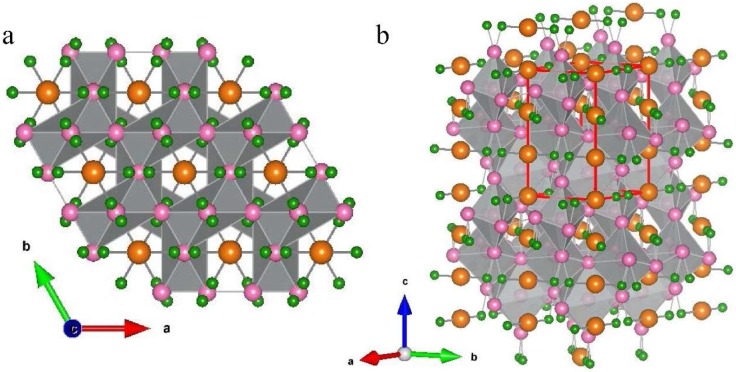
Crystal structure of MgNi_3_B_2_ (**a**) along the *c* axis highlighting the hexagonal array of B atoms in the Mg-B plane and (**b**) perspective view showing B atoms linked at apical equatorial sites of the Ni octahedral. Unit cell is outlined in red. Mg atoms are orange, B atoms are green, and Ni atoms are pink.

Prior to the definitive study by Manfrinetti et al. [5], reports on the composition and structure of the Mg-Ni-B phase synthesized under these conditions were inconsistent [14,15]. Proposed compositions ranged from Mg_2_Ni_5_B_4_ to MgNi_3_B_2_ and MgNiB [5]. Use of powder and single crystal diffraction techniques enabled clarification of the stoichiometry as MgNi_3_B_2_ with the structure shown in Figure 1. For MgNi_3_B_2_, atoms are ordered on specific sites with B arranged around Mg in a distorted hexagon as shown in Figure 1a looking down the *c* axis. Ni octahedral units are linked to B at both apical and equatorial sites of the Ni octahedra as shown in a perspective view in Figure 1b. In this structure, average B–B bond distances are 1.88 Å [5], consistent with typical bond lengths observed in diborides [16].

Production of boron compounds at lower temperatures than currently used in solid state synthesis is motivated by the potential for facile production at sample sizes that may scale to industrial use. This strategy has been demonstrated for MgB_2_ and includes the use of metal borohydrides to generate reactive gaseous boron species [17,18]. In this work, we utilise a useful attribute of NaBH_4_ powder when subjected to heating in the presence of specific metals [19]. This attribute is low temperature decomposition to form H_2_ and higher borohydride analogues catalysed by the presence of Mg and Ni [17,18]. Using an instrumented Parr-type reactor, we delineate the conditions for formation of MgNi_3_B_2_ and describe a possible mechanism for synthesis at temperatures above 670 °C.

## 2. Materials and Methods

### 2.1. Boride Synthesis

Molar ratios of Mg powder (<50 mesh size; 99.9% purity), Ni (<150 μm; 99.99% purity), and NaBH_4_ powder (99.99% purity) supplied by Sigma-Aldrich (Saint Louis, MO, USA) are weighed, ground in an agate mortar and placed into a 50 mL Parr reactor within a controlled atmosphere glove box containing Argon (99.99% purity). The reactor is designed with an internal fixed head and cylinder of Inconel 601 steel with a graphite seal to accommodate a maximum pressure of 20 MPa and a maximum average temperature of 725 °C at the base of the reactor. Prior to use, the reactor is thoroughly washed with water and dried in a vacuum oven up to 120 °C overnight. For some experiments, a boron nitride sleeve is introduced into the reactor to minimise reaction with the Ni-rich side-walls of the reactor.

The starting materials are added to the reactor, sealed tightly, and removed from the glove box. The reaction chamber is heated according to a standard protocol via thermocouple controller. The change in pressure is monitored during the reaction using a dial pressure gauge and an Ashcroft transducer mounted atop the reaction chamber. The temperature sensor is centred within the reactor and both temperature and pressure are recorded every minute. The temperature sensor is not embedded within the precursor materials which, at the start of synthesis, are located at the bottom of the chamber.

**Figure 2:**
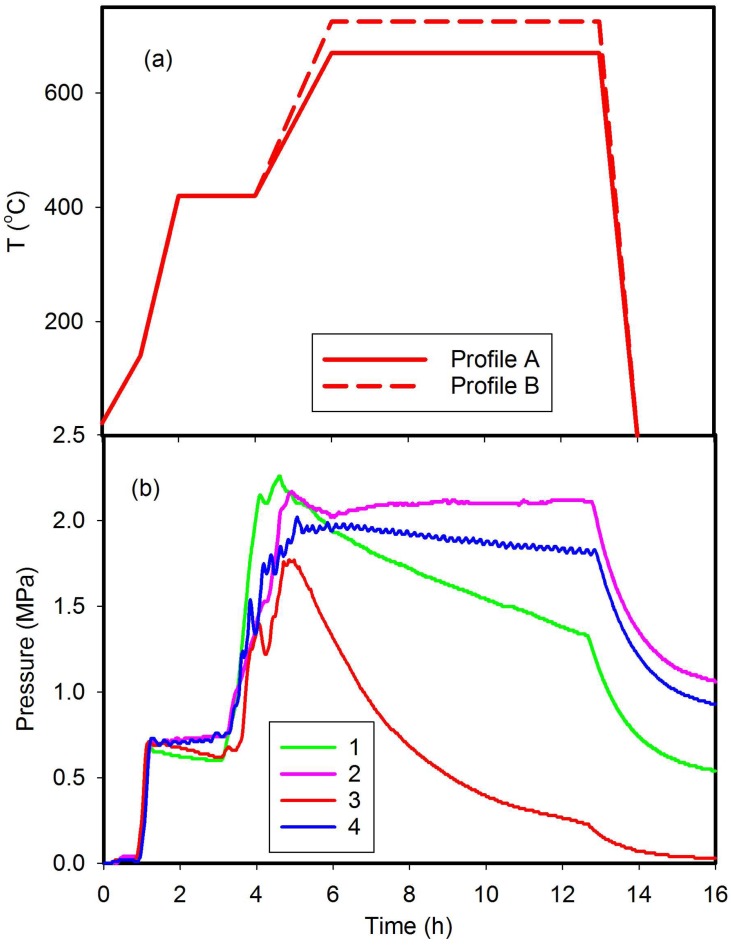
(**a**) Typical heating profile and (**b**) pressure profiles for synthesis of MgNi_3_B_2_ and other Ni-B phases with conditions as listed in Table 1.

The reactor design results in a thermal gradient between the bottom and top of the reactor during ramp up to the equilibration temperature. This gradient depends on the operating temperature recorded at the thermocouple and location within the reactor. For example, at a thermocouple reading of 500 °C at the centre of the reactor, the difference in temperature at the top or bottom of the reactor could be up to ±225 °C. The thermocouple measurements record average temperatures within the reactor and, as shown in Figure 2a, indicate progress of the reaction over time. This record provides an indirect measure of reaction stability (e.g., at equilibria; endothermic, exothermic) within a closed environment. An advantage of this reactor design is that gaseous phases such as sodium and sodium hydride condense at the cooler top of the reactor [18]. On cooling the reaction chamber to room temperature, the reactor is opened in the argon-filled glove box via slow pressure equilibration using a gas release valve.

A consistent heating rate of 10 °C/min is used in all reactions albeit at different temperatures, the heating rate is held constant for varying periods of time. In general, the reactor heating rate is held constant at selected base temperatures of: (i) ~140 °C; (ii) 420 °C; and (iii) 670 °C or 725 °C for variable periods of time. These specific heating rates and constant temperature periods are identified in Table 1, noting that T_max_ values are estimated for the base of the reactor.

### 2.2. Characterisation

Polycrystalline samples are characterized using X-ray powder diffraction and electron microscopy equipped with microanalysis. X-ray powder diffraction patterns are obtained using either Cu Kα1 or Co Kα1 radiation in Bragg Brentano geometry with 0.02° 2θ steps and a counting time of 10 s per step using PANalytical X-ray diffractometers. Diffraction patterns are refined and indexed using the software program Topas (Florence, KY, USA) [20]. X-ray diffraction patterns and electron microscopy indicate that all synthesised samples are multiphase with MgNi_3_B_2_ as a predominant phase. Samples with high proportions of MgNi_3_B_2_ (i.e., >80%) were selected for additional data collection using a PANalytical X-ray diffractometer (Almelo, The Netherlands) for subsequent Rietveld refinement using Topas. Schematic models of crystal structures shown in Figure 1 are built using data from Manfrinetti et al. [5] as input to the program VESTA [21].

Gases generated during reactions are collected for analysis at selected thermocouple temperatures of 65 °C and 120 °C using a Hamilton gas-tight syringe. These temperatures are correlated to base temperature of 120 °C and 200 °C, respectively. These gases are characterised using a Sercon 20–22 isotope mass spectrometer coupled with a GSL elemental analyser unit standardised to Ar and He. Additional gas analyses standardised for CO_2_, CH_4_ and N_2_O are undertaken with a Maestro MPS Headspace (Linthicum, MD, USA) and Agilent 7890A Gas Chromatograph (Santa Clara, CA, USA).

A Zeiss Sigma Field Emission SEM (Carl Zeiss Pty Ltd., North Ryde, Australia) equipped with an Oxford Instruments SDD detector (Abington, UK) is used for microscopy observations and energy dispersive spectroscopy (EDS) analysis. Samples are prepared for SEM/EDS by placing a thin layer of powder onto aluminium stubs with double-sided carbon tape. In general, samples are not coated with a conductive coating to avoid analytical interference(s). Elemental analysis is carried out at an accelerating voltage of 15 kV at 8.5 mm working distance. Excessively charging samples are imaged at lower accelerating voltages of 5 kV or 10 kV.

Quantitative elemental analyses are performed using a JEOL JXA 8530F field emission electron probe microanalyzer (FE-EPMA, JEOL, Tokyo, Japan) equipped with five wavelength-dispersive spectrometers (WDS) and using Probe for EPMA software (Eugene, OR, USA). For these analyses, powder samples are mixed with conductive resin and placed in a 30 mm diameter mould inside a hot mounting press. The sample mount is polished with a series of diamond pads and cloths to a mirror finish suited to electron microprobe analysis. Spot analyses on borides are performed using the following combined conditions: B and Mg Kα X-ray intensities are measured simultaneously at 7 kV accelerating voltage, followed immediately by intensity measurement of the Ni Kα X-ray line at 15 kV accelerating voltage. A focused beam is utilized and beam current maintained at 40 nA under both conditions.

For EPMA data reduction, the PROZA *φ*(*ρz*) matrix correction method of Bastin [22,23] is employed along with the MACJTA database of mass absorption coefficients. B Kα radiation is measured using an LDEB analyzing crystal (2d ~ 15nm) and an open detector slit (GFPC), while TAP and LIF analyzing crystals are used for Mg and Ni, respectively. An anti-contamination cold finger cooled by liquid nitrogen is used during all acquisitions. Standards include MgO, Ni_90_Fe_10_ alloy, and in-house MgB_2_. To avoid analytical errors associated with peak shift and peak shape changes for boron, the integrated intensity method for acquisition of full peak intensity is used in the *Probe for EPMA* software. The scan length for the boron peak corresponds to an energy range from ~160 eV to 270 eV with a counting time of 180 s for the integrated intensity measurement, followed by 60 s for background positions either side of the peak. An exponential fit to the background positions is used to model the background intensity under the peak. On and off-peak count times are 10 s for Ni and 20 s for Mg. Accompanying elemental X-ray maps of individual powder particles by WDS on the electron microprobe are conducted at 7 kV accelerating voltage, 40 nA beam current, and 20 ms dwell time in beam scanning mode.

**Figure 3:**
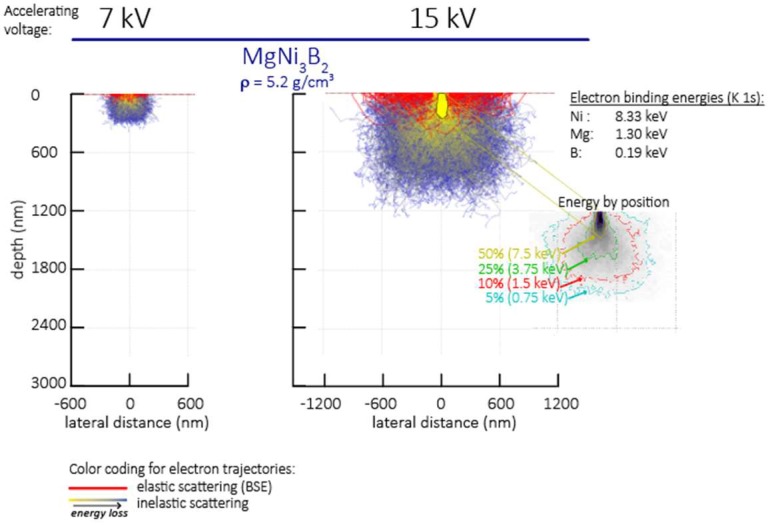
Electron beam–specimen interaction volumes for EPMA analyses at 7 kV and 15 kV accelerating voltage. The inset at right (“energy by position”) illustrates the amount of energy lost by the simulated electron trajectories as a function of depth and lateral distance. For example the Ni Kα X-ray line (K 1s binding energy 8.33 KeV) cannot be excited at depths greater than ~300 nm, whereas the excitation volume for B Kα (K 1s binding energy 0.19 keV) extends near the outermost limits of electron trajectories, exceeding 1 μm^3^ at 15 kV but confined to <<1 μm^3^ at 7 kV accelerating voltage.

In this study, the beam produced by a 7 kV and fully focused 40 nA source is chosen to reduce the electron beam-specimen interaction volume to <1 µm for boron and magnesium. The detection limits under the combined conditions are 0.16 wt % for B, 0.02 wt % for Mg, and 0.04 wt % for Ni. The average analytical error for boron for individual spot analyses is 8.7%. A statistical program is used to determine the envelope for production of characteristic X-rays using the EPMA under these operating conditions. At 7 kV the lateral and vertical spatial resolution for excitation of the Kα X-ray lines of B and Mg is <1 µm, as is that of the Ni Kα X-ray line at 15 kV as calculated using the CASINO Monte Carlo modeling program [24] for electron trajectories (Figure 3).

DC magnetization measurements are performed using a Cryogenics Ltd. Mini cryogen-free 5 T system (London, UK). Magnetization field isothermal loop is determined within ±5 T at 290 K.

## 3. Results

In general, data presented in this work are a summary of more than twenty-five separate experiments, including repeat syntheses, across a range of temperature and pressure conditions. Previous syntheses using metal borohydrides [17,18] have been used to guide the synthesis strategy for optimum production of MgNi_3_B_2_.

### 3.1. Synthesis

Figure 2a shows typical heating profiles that result in synthesis of MgNi_3_B_2_ using the 50 mL reactor including a heating profile for Run 1 listed in Table 1. The pressure profiles for reactions in Runs 1–4, which are most representative of this compositional suite, are shown in Figure 2b. In all reactions, pressure increases to a maximum value (P_max_) ranging between 1.7 MPa and 2.5 MPa when the reactor is at the maximum temperature (T_max_). The rate of pressure decrease after P_max_ is achieved varies for each reaction listed in Table 1, and is dependent on a number of variables including the ratio of starting materials within the reactor.

In all cases, MgNi_3_B_2_ is the major phase based on X-ray diffraction data and Rietveld refinement using Topas software. An excess of NaBH_4_ in the starting mixture results in a higher percentage of Ni-B phases and a lower percentage of MgNi_3_B_2_ (see Table 1). Table 1 describes generic variables that affect the relative proportions of MgNi_3_B_2_ product. For example, a higher T_max_ value of 725 °C at the base results in a higher yield of MgNi_3_B_2_ for the same molar ratio of starting materials and similar levels of autogenous pressure (i.e., Run numbers 1 and 2). Similarly, for T_max_ = 670 °C, a higher autogenous pressure may also result in a higher yield of MgNi_3_B_2_ (i.e., Runs 3 and 4). In general, the optimum base temperature and pressure range for highest yield of MgNi_3_B_2_ is between 670 °C–725 °C and 2.0 MPa–2.2 MPa.

### 3.2. Structure Determination

**Figure 4:**
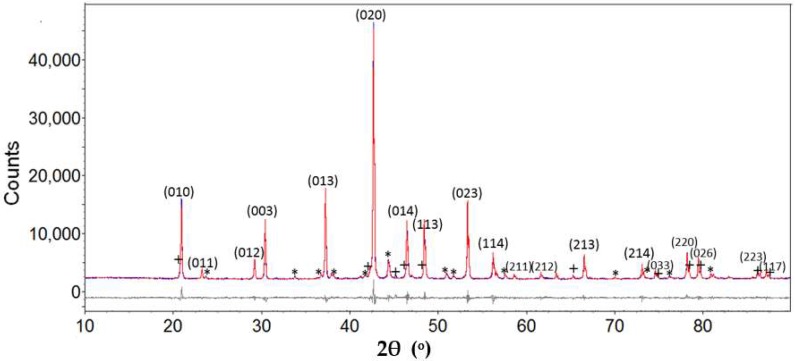
The experimental (blue), fitted (red), and difference (grey line below observed and calculated pattern) XRD profile for sample from Run 1. Indexed reflections are for MgNi_3_B_2_. Other phases including MgNi_6.7_B_2_ and MgNi_7_B_3_ denoted with * and +, respectively.

Powder diffraction patterns from selected samples listed in Table 1 are re-collected at a higher resolution using Cu Kα radiation for counting times suited to Rietveld refinement using TOPAS. Structural data from Manfrinetti et al. [5] are used as input to refinement of XRD data for these samples. Table 2 shows refined data for MgNi_3_B_2_ from this study compared with that by Manfrinetti et al. [5] based on data collected from a single crystal. Refined lattice parameters on the MgNi_3_B_2_ phase from this study are in good agreement with results reported by Manfrinetti et al. [5]. The resulting diffraction profile after Rietveld refinement of the Run 1 sample is shown in Figure 4. Phase analysis of this diffraction data also shows minor presence of other compounds including MgNi_6.7_B_2_ (7%) and MgNi_7_B_3_, (2%) with lower amounts of Ni-B phases.

### 3.3. Morphology and Microstructure

**Figure 5:**
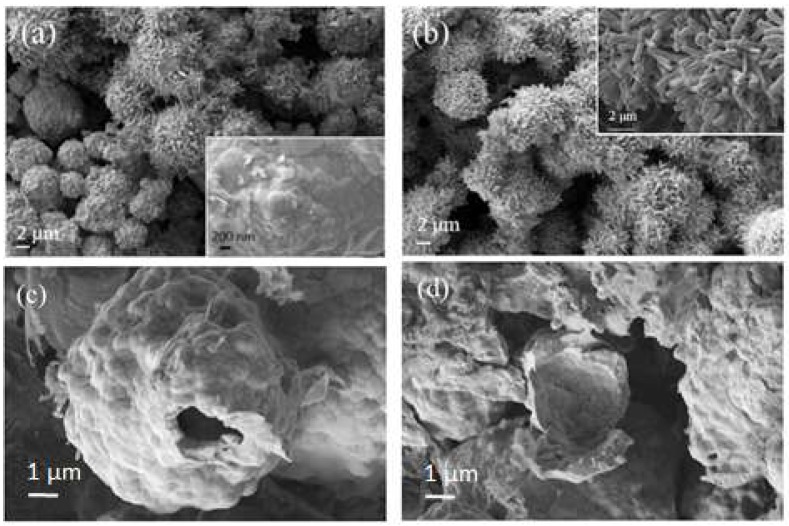
SEM images of MgNi_3_B_2_ powder produced by autogenous pressure reactions listed in Table 1. Higher resolution SEM images (inset) show: (**a**) cauliflower shaped morphology consisting of smoothly rounded crystals, (**b**) hexagonal rod-like crystals of flower-like morphology. (**c**,**d**) SEM images of as-prepared powder from Run 5 showing a Mg-Ni-B shell and partial, or broken, shells without a Ni-B core.

Representative SEM images of MgNi_3_B_2_ powder obtained from Run 1 are shown in Figure 5. Two different morphologies—one resembling a flower and the other a cauliflower—are common forms in all samples examined from these syntheses. Close observation of individual morphologies in Figure 5 reveals that a single flower comprises hexagonal rod-like crystals <200 nm diameter with lengths ranging between 1 μm and 2 μm. The inset shows a higher magnification image of the cauliflower-shaped MgNi_3_B_2_ morphology. Smooth, rounded particles with sizes varying from 50 nm up to several hundred nm form micron-sized agglomerates as shown in Figure 5a (inset). Figure 5c,d are SEM images of as-prepared powder from Run 5 with aggregates of nanometre sized particles arranged as hollow Mg-Ni-B shells or as remnant broken outlines of partially spherical shells without Ni-B cores.

**Figure 6:**
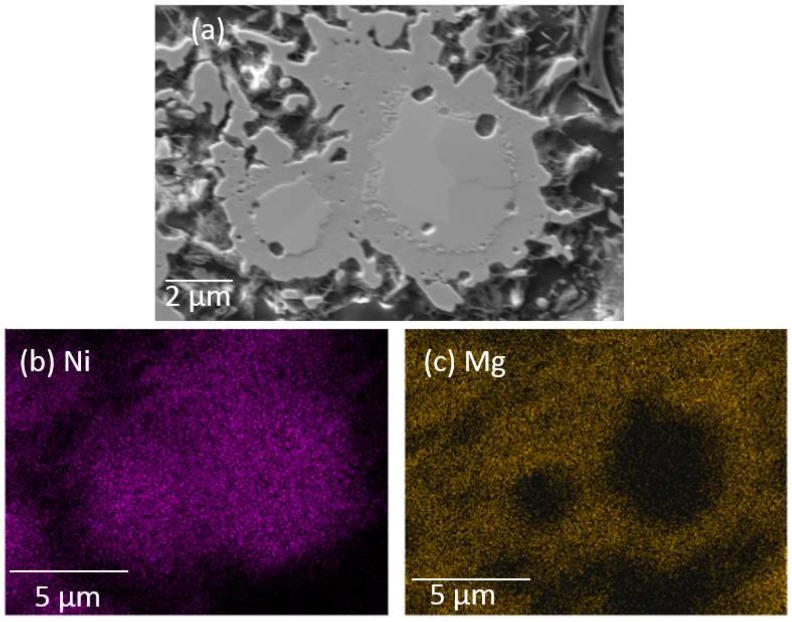
(**a**) Secondary electron image and elemental map using the FE-SEM for (**b**) Ni and (**c**) Mg of a polished sample from Run 3.

A polished section of MgNi_3_B_2_ grains produced from Run 3 is shown in Figure 6. The SEM image shows a ~15 μm × ~10 μm aggregate with different image contrast between the core and outer regions (Figure 6a). Figure 6b,c show the distribution of Ni and Mg within this aggregate using X-ray mapping. Ni is evenly distributed within the aggregate but it is clear that Mg is concentrated in the outer rim (Figure 6c). For this particular analysis using the FeSEM, the total collection time to acquire a boron signal with sufficient statistical certainty is >10 h which, with stage drift, precludes superposition of the boron distribution on these images.

**Figure 7:**
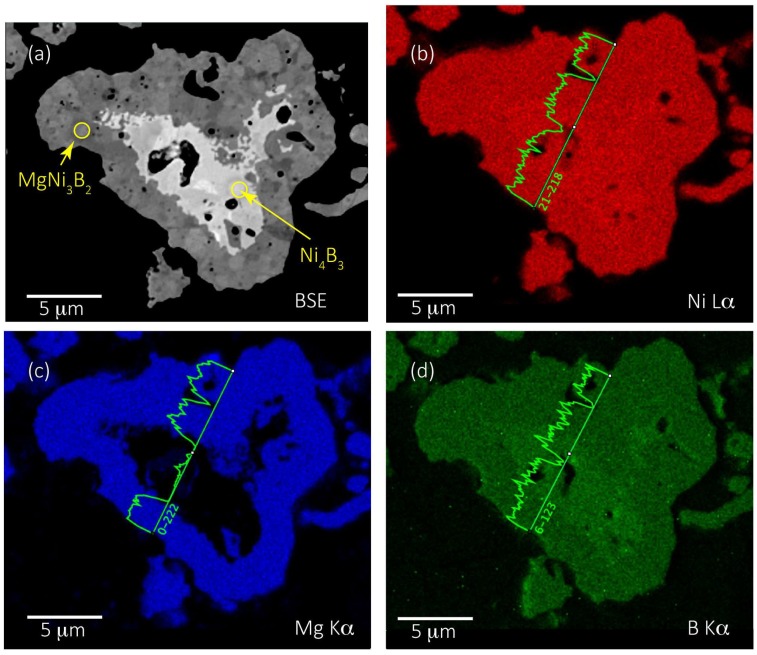
Aggregate from Run 5 showing (**a**) backscattered electron (BSE) image with image contrast corresponding to lighter Ni-B and darker Mg-Ni-B regions; and wavelength-dispersive spectrometers (WDS) X-ray maps of (**b**) Ni, (**c**) Mg and (**d**) B distribution in the aggregate.

A more precise method that also allows a high signal to noise ratio for low atomic number elemental X-ray mapping is EPMA. This method also enables quantitative spot analyses with a spatial resolution of 1 µm or less (see Section 2.2) of polished samples. In this work, we selected Mg, Ni, and B for analysis using WDS. An example of X-ray mapping by WDS for an aggregate from Run 5 is shown in Figure 7. Figure 7a is a backscattered electron image of the aggregate with accompanying element distributions for Mg, Ni, and B in Figure 7b–d. The backscattered electron image shows a wide variation in image contrast typically ascribed to variations in relative mean atomic number. In general, lighter regions in a backscattered electron image are higher in atomic number than darker regions [25].

The X-ray maps and detailed line scans in Figure 7b–d show that Ni and B are concentrated in the centre, or core, of the aggregate while all three elements are present in the outer margins. Mg is absent in the core of this aggregate. The map and line scan in Figure 7d show an increase in relative B concentration in the Ni-rich core region. This microstructure, that is, a core of Ni-B and an outer margin of Mg-Ni-B, is a common attribute of grains analysed from each of the syntheses listed in Table 1. Close inspection of Figure 7a also indicates a nuanced variation in image contrast within the outer margin of the aggregate. This characteristic is due to variations in orientation of the individual MgNi_3_B_2_ crystals that comprise the aggregate.

**Figure 8:**
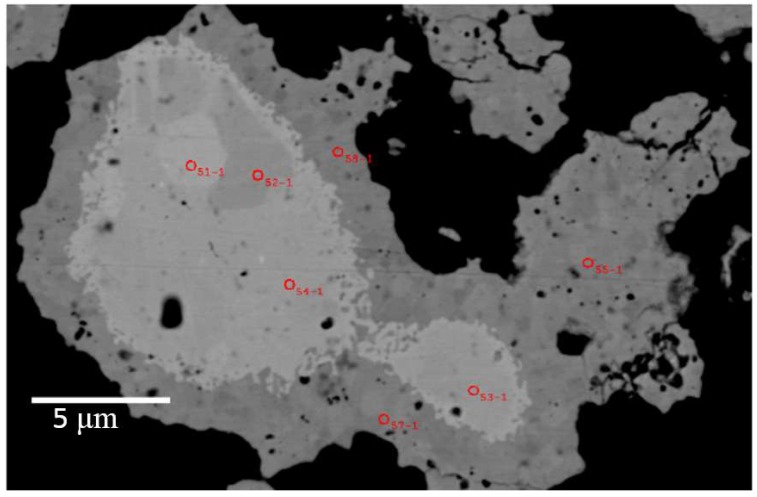
Backscattered electron image of an aggregate from Run 3 showing a bright core and darker outer margin corresponding to Ni-B rich and Mg-Ni-B rich phases, respectively. Red circles denote locations of spot analyses using the field emission electron probe microanalyzer (EPMA); average values for these spot analyses are listed in Table 3.

Figure 8 is a backscattered electron image of an aggregate from Run 3 (Table 1) showing a lighter core region and an outer, darker margin as well as locations of spot analyses using the EPMA. Again, the lighter region shows high levels of Ni and B and minimal or no Mg. Variations in crystal orientation are discernible in the Mg-rich outer margins of this aggregate from Run 3.

Table 3 provides average compositions measured by electron microprobe of selected spots in different aggregates from Runs 3 and 5. The number of point analyses for designated regions of the aggregates are listed in Table 3. These data show that the concentration of Mg in many core regions of aggregates is below detection under the given conditions. Nevertheless, the concentration of boron ranges between 5.53 wt % and 12.2 wt %, and the concentration of nickel between 87.2 wt % and 93.5 wt % in the cores of aggregates.

As shown in Table 3, the stoichiometry of Ni-B phases within the core regions includes Ni_2_B, Ni_3_B, and less commonly Ni_4_B_3_. In the outer margin, or shell regions of these aggregates, the concentration of magnesium, nickel, and boron is ~11.5 wt %, ~79 wt %, and 9 wt %, respectively. The stoichiometry of this outer margin is MgNi_3_B_2_, consistent with X-ray diffraction data. In Run 5, aggregates show a core region with some Mg content (Table 3; Run 5, “core 1”). The consistency of analyses, judged not only by the total element weight percent but also by the low standard deviations per element, suggests that this composition, MgNi_10.5_B_3_, may be an additional phase within the Mg-Ni-B compositional suite.

**Figure 9:**
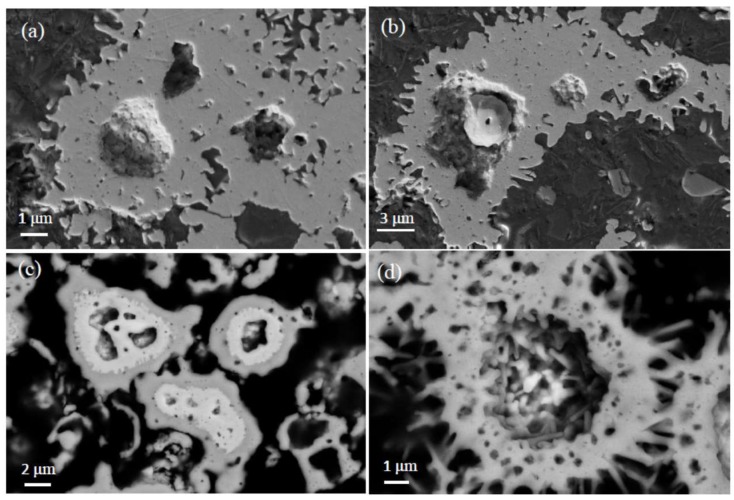
SEM image of the polished surface of reaction products from Run 5. In (**a**) a mound is apparent at the core part of the sample, while in (**b**) the mound shows a region in which material appears to have been removed. (**c**) Backscattered electron image shows the structure of a Ni-B core region surrounded by a MgNi_3_B_2_ rim. (**d**) Backscattered electron image of Mg-Ni-B particle showing core region that is presumably removed through sample preparation.

Figure 9a,b show secondary electron images of polished reaction product from Run 5. In these examples, the core region appears as an elevated mound which in one case is intact, while in the other (Figure 9b), part of the core region is removed. Figure 9c,d are backscattered electron images of the sample from Run 5. The Mg-Ni-B shell with Ni-B core structure is evident for this sample after a reaction time of 40 h. The backscattered electron image in Figure 9d again shows a core region that is removed from the Mg-Ni-B aggregate, presumably via the sample polishing step.

**Figure 10:**
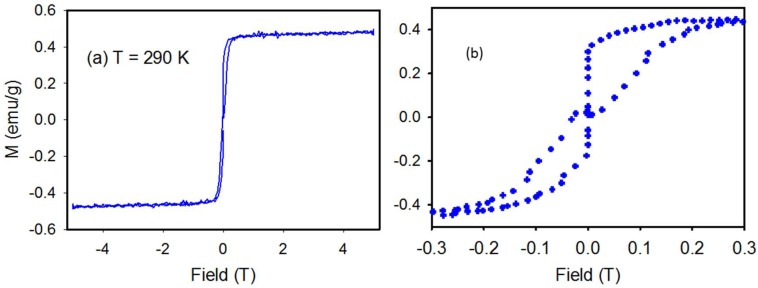
Magnetic data for Run 4 sample showing (**a**) Field dependant magnetization at 290 K. (**b**) Higher magnification view of magnetization at low field.

Figure 10a shows a magnetic hysteresis loop at 290 K measured over a ±5 T field for product from Run 4. The magnetization increases with increasing magnetic field and then saturates at 0.2 T with a saturation value of 0.45 emu/g. Figure 10b shows the relatively narrow hysteresis loop with a coercivity field of 250 Oe.

## 4. Discussion

Interest in Mg-Ni and Mg-Ni-B compounds is predominantly associated with their potential for use in Ni-metal hydride batteries [26,27] and for hydrogen storage [28,29]. In this latter instance, addition of MgNi_3_B_2_ to a metal hydride system (e.g., LiBH_4_-MgH_2_) provides a catalytic effect that increases hydrogen storage by ~9% [28]. Further work [29,30] shows that addition of Ni-B to the same system also enables growth of MgNi_3_B_2_ in the dehydriding process. Thus, while MgNi_3_B_2_ appears to be an ineffective material for hydrogen storage *per se* [14], the phase may be important in the evolution of effective hydrogen storage technology. Other compositions within the Mg-Ni-B phase space are also of interest, in particular MgNi_7_B_3_ [4] and a new ternary diboride suite with compositions Mg_3+x_Ni_7-x_B_2_ [31]. This new diboride suite is also amenable to metal doping with consequent influence on magnetic properties [31]. In this discussion, we focus on MgNi_3_B_2_ and other compounds formed under autogenous pressure conditions.

### 4.1. Synthesis of MgNi_3_B_2_

In earlier work, we described an approach to synthesise MgB_2_ using autogenous pressure generated by the decomposition of NaBH_4_ or KBH_4_ [17,18] in the same type of reactor as in this study. In this earlier work we inferred, on the basis of pressure–temperature data [18], that in the presence of Mg, NaBH_4_ begins to decompose at ~80 °C–120 °C resulting in the formation of H_2_ (g) and B_2_H_6_ (g) and higher order borohydrides as noted by earlier researchers [19,32]. We also inferred higher temperature gas–solid reactions in this type of reactor under a range of conditions [17,18]. In these earlier studies, we used the thermocouple reading at the centre of the reactor to estimate the temperatures of these reactions. In this work, we have recalibrated the reactor to provide an estimate of the temperature experienced by the starting materials at the base of the reactor, in particular, at the hold temperatures used in all experiments and using the same ramp rates.

Puszkiel and Gennari note that alkali metal borohydrides do not desorb hydrogen below ~400 °C [30]. However, our earlier work [17,18] and analyses of gases produced during the reactions shown in Table 1 suggest otherwise. Accordingly, our analyses of gases sampled when the reactor thermocouple readings are 65 °C and 120 °C show the presence of H_2_ at 5% and 15% content, respectively. Our calibration of the thermocouple reading with the measured temperature at the base of the reactor suggests that NaBH_4_ experiences maximum temperatures between 120 °C and 200 °C, respectively, at each of these gas sampling points. Thus, with no other source for H_2_ gas, we conclude that this is derived from the initial production of B_2_H_6_ via catalytic decomposition of NaBH_4_ [19,32].

As a further check on gas composition, NO_2_ gas concentrations are measured at 0.03 ppm and 0.05 ppm while CO_2_ is 83 ppm and 127 ppm for the 120 °C and 200 °C samples, respectively. Methane is measured at 3.83% and 1.97% for the 120 °C and 200 °C samples, respectively. For gas analyses in this work, an additional signal shows a mass number equivalent to B_2_H_6_ is also present. This mass number may be equivalent to O_2_ albeit under all conditions the opportunity for gas leakage into or out of the sample container is minimal, as indicated by the NO_2_ and CO_2_ gas concentrations. Nevertheless, our analytical tools are not explicitly benchmarked against a standard B_2_H_6_ aliquot. Hence, we infer that B_2_H_6_ is also present in the reactor due to the catalytic decomposition of NaBH_4_.

The increase in pressure up to and including T ∼ 420 °C as shown in Figure 2 is primarily due to the evolution of H_2_ and B_2_H_6_ and the formation of higher borohydrides [17]. Figure 3 shows an increase in reactor pressure as the thermocouple reading increases up to 420 °C at which the ramp rate is held for 20 min. With continued heating, the pressure decreases slightly due to the formation of higher order diborane species [33] or hydrides such as MgH_2_ [4,34]. MgH_2_ nanofibres form via reaction with Mg and H_2_ gas at a pressure of 1 MPa to 2.5 MPa in the range 420 °C < T < 725 °C [34] and also form under similar autogenous pressure conditions with the reaction between Mg and NaBH_4_ [18]. Decomposition of NaBH_4_ is accelerated in the presence of Ni and Mg as well as by intermediate phases such as MgH_2_ [18,35]. The hydride, Mg_2_NiH_4_, also forms via the mechanical milling reaction of MgH_2_ with Ni at temperatures >400 °C and pressures >8 MPa [36,37]. As noted in earlier work, formation of MgH_2_ is a likely intermediate step during heating of the reactor to >420 °C [18]. This intermediate phase enables formation of other Mg-Ni, Mg-B, Ni-B, and Mg-Ni-B compounds at higher temperatures [17].

In this process, a range of competing reactions—both solid–liquid, solid–gas, and liquid–gas—occur as the temperature is increased to T_max_. At ~420 °C, the formation of MgH_2_ and further decomposition of NaBH_4_ and higher order diboranes occurs [18]. In addition, as T > 500 °C other intermediate phases such as Mg(BH_4_)_2_, NaMgH_3_, and MgB_2_ are likely to form [17]. We have also explored the reactions of Ni metal with NaBH_4_ under similar conditions and note that Ni-B compounds form at autogenous pressures ranging between 1 MPa and 2 MPa and thermocouple temperatures of 500 °C < T < 725 °C (unpublished data). At these higher temperatures in the presence of H_2_ and borohydride gasses, we suggest that Mg-Ni hydrides and Mg-Ni borides form. Other likely phases include alkali metal composites similar to that described by Shao et al. [29] which include LiH-MgB_2_ with Ni-B particles. The work by Shao et al. [29] shows that MgNi_3_B_2_ forms as hydrogen is removed from the composite at 400 °C. For the reactions described here, we suggest that a NaH-MgB_2_ composite with Ni-B forms, and enhances the production of MgNi_3_B_2_.

With the increase in temperature to T_max_, and a hold for specific periods of time, vapour phase transfer of elements and compounds is accelerated and also results in condensation of some compounds and elements at the cooler top of the reactor [18]. For example, loss of H_2_ from the immediate vicinity of the reactants can occur through volatilisation of NaH and/or Na as well as residual MgH_2_ that subsequently condenses at the top of the reaction chamber [17,18]. At these higher reactor temperatures (i.e., >500 °C), the presence of Na as a liquid or as a gas is likely [18]. In addition, the decomposition of NaBH_4_ is accelerated in the presence of MgH_2_ [38]. Further complexity arises because, between 500 °C and 600 °C, Mg(BH_4_)_2_ decomposes to form MgH_2_ [17,39]. Our analysis of MgB_2_ formation using an autogenous pressure reaction between Mg and NaBH_4_ shows that Na is poorly soluble in the diboride structure and thus, is not detected in the final product [17]. Under these reactor conditions where the base may reach up to 650 °C [18], Na is most commonly observed in phases at the cooler top of the reactor. EPMA data for MgNi_3_B_2_ produced in this work suggests that Na is also not soluble in the predominant product.

Thus, reactions involving solid–gas and liquid–gas interactions compete at higher temperatures as illustrated by the change in reactor pressure(s) shown in Figure 2b. As new stable phases form, the autogenous pressure in the reactor changes over time (Figure 2b). In general, we interpret that gas phase reactions are near complete if the autogenous pressure is low (i.e., < 0.1 MPa) when the reactor is cooled from T_max_ (e.g., Run 3 of Figure 2b). Given the complexity of these intermediate reactions, the formation of MgNi_3_B_2_ is nevertheless governed by generic conditions such as availability of elements (e.g., Mg, Ni and B) in appropriate ratios (or compounds) and the temperature and pressure of reactions. We suggest that the overall reaction for formation of MgNi_3_B_2_ in this system is:

Mg + 3Ni + 2NaBH_4_ = MgNi_3_B_2_ + 2NaH + 3H_2_ (1)

The additional products in Equation (1)—NaH and H_2_—are both gas phase at T_max_. Hence, it is reasonable to assume that microstructures for MgNi_3_B_2_ are moderated by solid–gas, liquid–gas, and possibly, vapour–liquid–solid mechanisms.

As noted in Table 1 and shown in Figure 4, other Mg-Ni phases occur in minor amounts as part of this synthesis method. For example, MgNi_7_B_3_ and MgNi_6.7_B_2_ occur as minor products in all experiments shown in Table 1. We also explored this method to produce, for example, MgNi_7_B_3_ in higher yields under similar conditions with appropriate adjustment for molar ratios of starting materials. A maximum yield of ~40% for MgNi_7_B_3_ is obtained at T_max_ = 450 °C at the thermocouple and ~4 MPa pressure for 8 h. Optimum yields occur when Mg is in excess by 20–30%, consistent with the practice noted by Liao et al. [4].

### 4.2. Microstructures

SEM images of powders formed by this process show aggregates containing many nanometre-scale grains with a range of morphologies (Figure 5). A first impression from Figure 5 and the XRD data suggests that the aggregates are comprised of a single phase Mg-Ni-B compound with grains of varying size and shape. However, the microstructures revealed by polished sections provide observations that hint at a different interpretation and at key steps in the formation of MgNi_3_B_2_ aggregates by this synthesis method.

For example, the centres of most aggregates contain a Ni-boride phase such as Ni_2_B or Ni_3_B as shown in Table 3 and Figure 7, Figure 8 and Figure 9. The higher boron content Ni_4_B_3_ is also present and usually a phase that lies between the Ni-rich core and the MgNi_3_B_2_ outer regions of an aggregate. This morphology suggests that Ni-rich phases formed first in this process presumably via the interaction of H_2_ (g) and B_2_H_6_ (g) with Ni metal at T ≤ 420 °C. The autogenous pressure at low temperatures in the reactor is insufficient to ensure H_2_ diffusion into bulk Ni albeit H_2_ may penetrate surface layers of individual grains to ~30 µm [40]. Thus, it is likely that Ni-borides form at lower temperatures through reaction with B_2_H_6_ and the higher order analogues as shown in Reactions (2) and (3)

4Ni + B_2_H_6_ = 2Ni_2_B + 3H_2_ (2)

6Ni + B_2_H_6_ = 2Ni_3_B + 3H_2_ (3)

Above 420 °C, with formation of MgH_2_ and Mg(BH_4_)_2_, their subsequent decomposition [17,40], and the continued decomposition of NaBH_4_ [38], reactive Mg and B species may result that enhance formation of Mg-Ni and Mg-Ni-B phases. MgB_2_ also forms above 350 °C under these autogenous pressure conditions [18] but is not evident in the final product due to formation of MgNi_3_B_2_ [29,30].

For the experiments listed in Table 1, the evidence from XRD data does not correlate well with the notional abundance of the Ni-boride phases shown via polished section microscopy. We attribute the low estimated values for Ni-boride phases using XRD Rietveld refinement to the modest signal/noise ratio, few reflections due to symmetry and overlap of key reflections for MgNi_3_B_2_ and MgNi_6.7_B_2_ [4,5] as well as with Ni_2_B and Ni_3_B. This suggestion also implies that the relative proportions of phases in the products shown in Table 1 are not accurate and, we estimate, may be in error by as much as 10% relative. An intense X-ray beam (e.g., rotating anode; synchrotron) is required to provide higher accuracy of calculations for relative proportions of phases in these aggregates.

The relationships between the inner and outer parts of an aggregate are varied in detail. In general, the core region is a boron-poor Ni-boride with transition to boron-rich phases (e.g., Ni_4_B_3_; MgNi_3_B_2_) towards the outer margin, or shell, of an aggregate. This general trend also supports the suggestion that Mg-rich phase(s) form after the Ni-borides through solid–gas and/or liquid–gas reactions. While we have no definitive evidence, the formation of MgNi_3_B_2_ rods or needles (Figure 5b) may occur via a vapour-–liquid–solid mechanism in this type of environment. The presence of “vacant” cores—or an empty shell morphology—suggests that in some cases, gases such as H_2_ are trapped within the aggregate. Through a change in reactor pressure (with change in temperature), the trapped gas exits the core of the aggregate. The gas is likely to be trapped during a rapid growth stage when the reactor temperature is increased to T_max_.

The consistent composition of the aggregate shell region as demonstrated by EPMA analyses in Table 3 is noteworthy. Nevertheless, two sets of spot analyses in Table 3 show that other Mg-Ni-B phases may co-exist and deserve further attention. These analyses are for aggregates from Run 5 for which the T_max_ hold time is 40 h. The inner region of one aggregate shows an average composition of Mg_0.34_Ni_3.49_B (or ~MgNi_10_B_3_) which has not been observed previously [4,5,31]. The outer regions of an aggregate also from Run 5 shows a composition—at one location only—of MgNi_4_B_2_. These compositions are significantly different to the notional stoichiometry of the starting materials. With different reactor conditions (e.g., longer reaction times, higher proportion of Ni and B in starting materials), new forms of Mg-Ni-B phases such as those implied by the EPMA data in Table 3 may be obtained.

### 4.3. Magnetic Properties

Manfrinetti et al. [5] present magnetic properties of polycrystalline MgNi_3_B_2_ with an applied field of 0.7 Tesla. At room temperature, MgNi_3_B_2_ is diamagnetic [5] consistent with filled Ni-3d bands and MgNi_3_B_2_ becomes positive at ~165 K. In contrast, the sample from Run 4 of this study shows substantially different magnetic behaviour. Figure 10a shows a magnetic hysteresis loop at 290 K for Run 4 where magnetization increases with increasing magnetic field and then saturates at 0.2 T with a saturation value of 0.45 emu/g. The relatively narrow hysteresis loop with a coercivity field of 250 Oe may be due to the presence of a ferromagnetic impurity such as elemental nickel. This distinctly different magnetic signature of multi-phase MgNi_3_B_2_ aggregates produced by autogenous pressure synthesis is clearly influenced by the core-shell nature of the product in which a Ni-boride core is encompassed by nano-scale MgNi_3_B_2_ rods or particles.

## 5. Conclusions

In summary, the ternary intermetallic MgNi_3_B_2_ as aggregates in conjunction with Ni-boride phases have been synthesized using autogenous pressure by the reaction of NaBH_4_, Mg, and Ni powder. XRD analysis of the primary product is consistent with previous single crystal and powder diffraction studies of MgNi_3_B_2_ by Manfrinetti et al [5]. However, microanalyses of polished sections show that these micrometer scale aggregates contain inner cores of Ni-boride phases such as Ni_2_B and Ni_3_B surrounded by successively more boron-rich phases such as Ni_4_B_3_ and MgNi_3_B_2_. In this synthesis method, solid–gas and liquid–gas reactions are dominant formation mechanisms for intermediate phases and final products. The microstructures also indicate that Ni-borides form at lower temperature than MgNi_3_B_2_ in this process. EPMA data provide circumstantial evidence for other Mg-Ni-B phases such as MgNi_10.5_B_3_, and possibly, MgNi_4_B_2_. These core-shell structures of Ni-boride/MgNi_3_B_2_ aggregates demonstrate substantially different magnetic properties to stoichiometric MgNi_3_B_2_ that is synthesized only at higher temperatures and longer time intervals [5].

## Figures and Tables

**Table 1:** Synthesis conditions * for MgNi_3_B_2_.

Run No.	NaBH_4_ (g)	T_max_^£^ (°C)	t_Tm_ (h)	P_max_ MPa	% MgNi_3_B_2_	Other Phases **
1	0.76	725	8	2.2	88%	MgNi_6.7_B_2_ MgNi_7_B_3_
2	0.76	670	8	2.1	70%	MgNi_6.7_B_2_ MgNi_7_B_3_ Ni_2_B Ni_3_B
3	0.72	670	8	1.7	76%	MgNi_6.7_B_2_ MgNi_7_B_3_ Ni_2_B Ni_3_B
4	0.72	670	8	2.0	90%	MgNi_6.7_B_2_ Ni_2_B MgNi_7_B_3_
5	0.72	670	40	2.0	85%	MgNi_6.7_B_2_ MgNi_7_B_3_ Ni_3_B
6	0.72	725	30	2.5	76%	Ni_2_B (12%) MgNi_7_B_3_ (11%)

* Molar ratio of Mg:Ni is 1:3 for all reactions; ** other phases are generally less than 10% by Rietveld refinement except where noted in parentheses. t_Tm_ is the time the reactor is held at T_max_; ^£^ Base temperature.

**Table 2:** Results of refinement for MgNi_3_B_2_ from Run 1.

Parameter	MgNi_3_B_2_ (This Work)	MgNi_3_B_2_ [5] (Single Crystal)
Space group	P6_4_22	P6_4_22
a (Å)	4.8799 (1)	4.8800 (1)
c (Å)	8.7884 (2)	8.7870 (1)
Cell volume (Å^3^)	181.2 (1)	181.22
R_wp_	3.8%	4.0%
R_p_	2.7%	1.9%
GoF	1.98	0.93
Atom: Ni_2_ (z; 6f)	0.2062 (1)	0.2083 (1)
Atom: B (x; 6i)	0.6102 (7)	0.6110 (8)

**Table 3:** Average compositions * (±1 σ) of selected regions in aggregates from Runs 3 and 5.

		Element (wt %)				Stoichiometry			
Compound	n ^+^	B	Mg	Ni	Total	B	Mg	Ni	Total
Run 3									
Ni_2_B (core 1)	4	8.36 (0.47)	0.04 (0.01)	89.8 (0.9)	98.2 (1.3)	1	0.00 (0.00)	1.98 (0.10)	2.98 (0.10)
Ni_3_B (core 2)	5	5.58 (0.32)	0.03 (0.01)	93.5 (1.3)	99.1 (1.5)	1	0.00 (0.00)	3.09 (0.17)	4.09 (0.17)
Ni_4_B_3_ (mid-core)	5	12.0 (0.1)	0.04 (0.02)	87.9 (0.5)	99.9 (0.4)	3	0.00 (0.00)	4.06 (0.06)	7.06 (0.06)
MgNi_3_B_2_ (shell)	5	9.01 (0.34)	11.5 (0.1)	79.2 (1.4)	99.6 (1.3)	2	1.13 (0.05)	3.24 (0.16)	6.37 (0.21)
Run 5									
MgNi_10.5_B_3_ (core1)	3	4.76 (0.11)	3.65 (0.37)	90.2 (0.4)	98.6 (0.1)	3	1.02 (0.04)	10.50 (0.09)	14.52 (0.10)
Ni_3_B (core2)	2	5.53	bdl	93.2	98.7	1	bdl **	3.11	4.11
Ni_4_B_3_ (mid-core)	1	12.2	bdl	87.2	99.5	3	bdl **	3.94	6.94
MgNi_3_B_2_ (shell)	8	9.27 (0.14)	11.7 (0.1)	78.1 (1.0)	99.1 (0.9)	2	1.12 (0.02)	3.10 (0.07)	6.22 (0.09)
MgNi_4_B_2_	1	7.83	8.73	84.1	100.7	2	0.99	3.96	6.95

* All measurements by electron probe microanalysis; ** bdl = below detection limit, ^+^ number of point analyses.
